# Invasive versus medically managed acute coronary syndromes with prior bypass (CABG-ACS): insights into the registry versus randomised trial populations

**DOI:** 10.1136/openhrt-2020-001453

**Published:** 2021-02-26

**Authors:** Matthew M Y Lee, Mark C Petrie, Paul Rocchiccioli, Joanne Simpson, Colette E Jackson, David S Corcoran, Kenneth Mangion, Ammani Brown, Pio Cialdella, Novalia P Sidik, Margaret B McEntegart, Aadil Shaukat, Alan P Rae, Stuart H M Hood, Eileen E Peat, Iain N Findlay, Clare L Murphy, Alistair J Cormack, Nikolay B Bukov, Kanarath P Balachandran, Ian Ford, Olivia Wu, Alex McConnachie, Sarah J E Barry, Colin Berry, the CABG-ACS Investigators

**Affiliations:** 1 Institute of Cardiovascular and Medical Sciences, University of Glasgow, Glasgow, UK; 2 West of Scotland Heart and Lung Centre, Golden Jubilee National Hospital, Clydebank, UK; 3 Cardiology, Glasgow Royal Infirmary, Glasgow, UK; 4 Cardiology, Western Infirmary, Glasgow, UK; 5 Cardiology, Royal Alexandra Hospital, Paisley, UK; 6 Cardiology, Royal Blackburn Hospital, Blackburn, UK; 7 Robertson Centre for Biostatistics, University of Glasgow, Glasgow, UK; 8 Health Economics and Health Technology Assessment, University of Glasgow, Glasgow, UK; 9 Department of Mathematics and Statistics, University of Strathclyde, Glasgow, UK

**Keywords:** coronary angiography, coronary artery bypass, acute coronary syndrome, myocardial infarction, outcome assessment, health care

## Abstract

**Background:**

Coronary artery bypass graft (CABG) patients are under-represented in acute coronary syndrome (ACS) trials. We compared characteristics and outcomes for patients who did and did not participate in a randomised trial of invasive versus non-invasive management (CABG-ACS).

**Methods:**

ACS patients with prior CABG in four hospitals were randomised to invasive or non-invasive management. Non-randomised patients entered a registry. Primary efficacy (composite of all-cause mortality, rehospitalisation for refractory ischaemia/angina, myocardial infarction (MI), heart failure) and safety outcomes (composite of bleeding, stroke, procedure-related MI, worsening renal function) were independently adjudicated.

**Results:**

Of 217 patients screened, 84 (39%) screenfailed, of whom 24 (29%) did not consent and 60 (71%) were ineligible. Of 133 (61%) eligible, 60 (mean±SD age, 71±9 years, 72% male) entered the trial and 73 (age, 72±10 years, 73% male) entered a registry (preferences: physician (79%), patient (38%), both (21%)).

Compared with trial participants, registry patients had more valve disease, lower haemoglobin, worse New York Heart Association class and higher frailty.

At baseline, invasive management was performed in 52% and 49% trial and registry patients, respectively, of whom 32% and 36% had percutaneous coronary intervention at baseline, respectively (p=0.800). After 2 years follow-up (694 (median, IQR 558–841) days), primary efficacy (43% trial vs 49% registry (HR 1.14, 95% CI 0.69 to 1.89)) and safety outcomes (28% trial vs 22% registry (HR 0.74, 95% CI 0.37 to 1.46)) were similar. EuroQol was lower in registry patients at 1 year.

**Conclusions:**

Compared with trial participants, registry participants had excess morbidity, but longer-term outcomes were similar.

**Trial registration number:**

NCT01895751.

**Key questions**

**What is already known about this subject?**

- Pivotal clinical trials of invasive management versus non-invasive medical management in acute coronary syndromes (ACS) excluded patients with prior coronary artery bypass graft (CABG).

**What does this study add?**

- The CABG-ACS trial and registry provides novel, contemporary insights into an understudied subgroup of ACS patients with a substantial health burden.

**How might this impact on clinical practice?**

- The CABG-ACS pilot trial and registry fills in an evidence gap on the natural history and optimal treatment strategy for this comparatively large subgroup of patients. Furthermore, the CABG-ACS trial and registry may be helpful in the design of clinical trials in this patient group.

## Introduction

Coronary artery bypass graft (CABG) surgery is standard of care for patients with obstructive coronary artery disease. However, occlusive disease of saphenous vein grafts is common within 10 years of surgery,^1–3^ meaning patients with prior CABG have a progressive longer-term risk of recurrent ischaemia, including angina and myocardial infarction (MI), heart failure (HF) and death. Given the large number of CABG recipients living globally, and their health complexities, including increasing age and multimorbidity, this represents an increasing healthcare challenge globally, not least because of rehospitalisation due to recurrent ischaemia.^4 5^ Chest pain is the most common reason for hospital admission in the UK and about 1 in 10–15 patients admitted to hospital with an acute non-ST segment elevation acute coronary syndrome (NSTE-ACS) have a prior CABG.^6^

Pivotal clinical trials comparing routine invasive management vs conservative non-invasive management in unstable coronary syndromes excluded patients with prior CABG (online supplemental table 1). Current guidelines recommend a routine early invasive strategy in higher risk NSTE-ACS patients.^7–9^ However, invasive management is performed less often in NSTE-ACS patients with prior CABG, probably because the risk:benefit balance is considered to be less favourable in these patients compared with those without prior CABG.^10–12^ Furthermore, when invasive management is performed, percutaneous coronary intervention (PCI) is less likely in prior CABG patients,^11–13^ implying a lower likelihood of benefit with a routine invasive strategy. Real-world evidence implies clinical practice departs from the results of systematic reviews and guidelines,^14 15^ indicating physician and patient preferences for treatment options may be relevant. Overall, evidence is lacking to inform the validity of current guideline recommendations,^7–9^ in NSTE-ACS patients with prior CABG. This important subgroup of ACS patients remains comparatively understudied. Enrolment into randomised trials can be challenging,^16^ particularly when the intervention disrupts standard care. Enrolment of elderly patients may be challenging, sometimes leading to premature trial discontinuation.^17–19^

10.1136/openhrt-2020-001453.supp1 Supplementary data

We hypothesised that the clinical characteristics, treatment and health outcomes would differ between participants enrolled in a randomised controlled trial of routine invasive versus routine non-invasive management in NSTE-ACS patients with prior CABG, and participants enrolled in the registry due to physician and/or patient preference. We aimed to prospectively gather information on the trial and registry participants in order to gain contemporary information on the natural history and reasons for trial participation or not.

## Methods

We undertook a randomised controlled trial of routine invasive management vs routine conservative management in NSTE-ACS patients with prior CABG. Concurrently, patients who were ineligible for randomisation and who gave informed consent were entered into an observational registry. The study design,^20^ and results of the main trial have been published.^21^

### Study population

Inclusion criteria were: (1) unstable angina or non-ST segment elevation MI; (2) stabilised symptoms without recurrent chest pain or intravenous therapy for 12 hours and (3) prior CABG.

Exclusion criteria were: (1) refractory ischaemia (ie, recurrent angina with minimal exertion or at rest (ie, Canadian Cardiovascular Society (CCS) class III or IV) not controlled by medical therapy); (2) cardiogenic shock; (3) lack of informed consent and (4) unsuitable for invasive management.

### Randomisation

**Figure 1:**
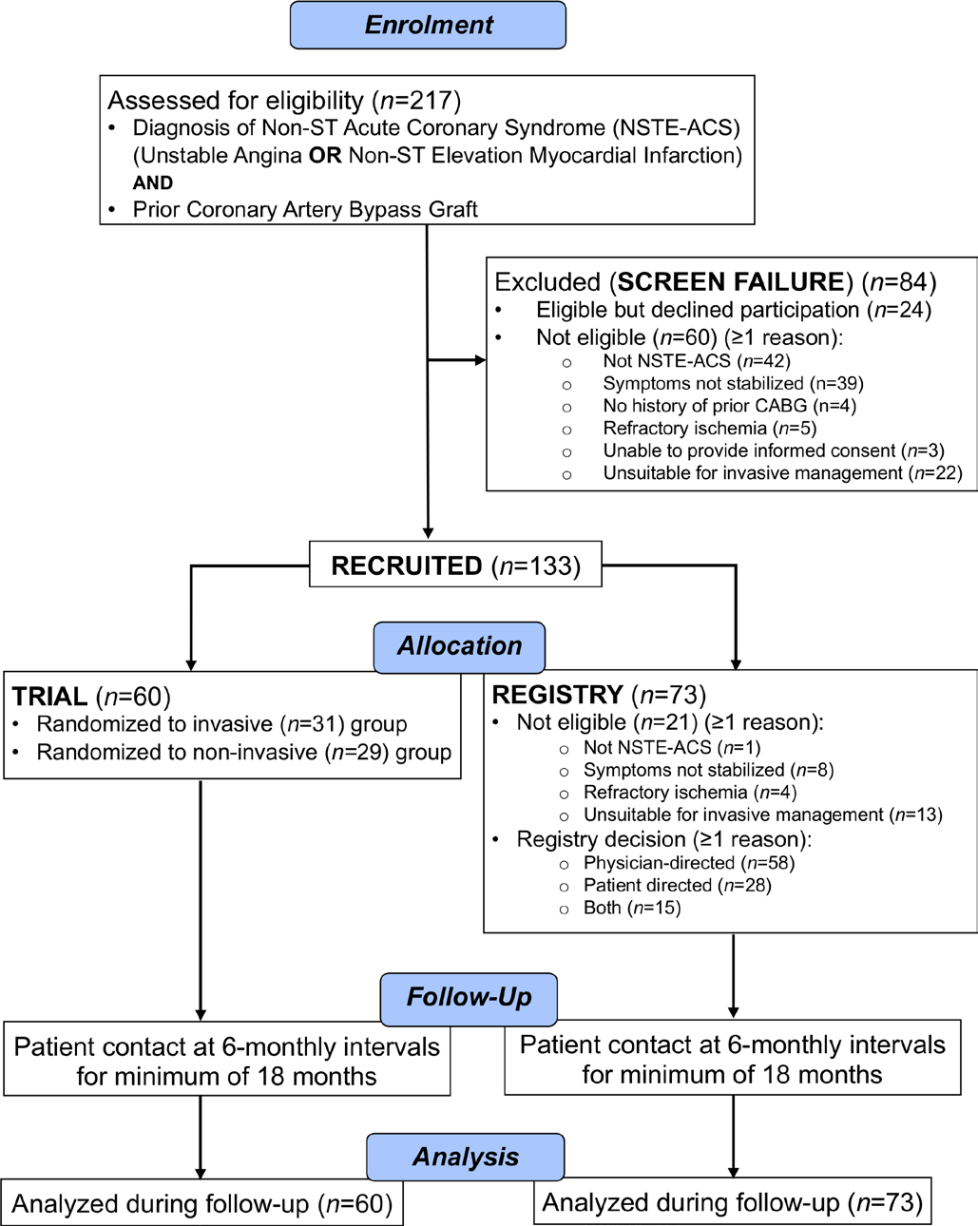
CONSORT diagram in CABG-ACS. CABG, coronary artery bypass graft; CONSORT, Consolidated Standards of Reporting Trials; NSTE-ACS, non-ST segment elevation acute coronary syndrome.

Patients fulfilling inclusion criteria without any exclusion criteria who consented to participate in the randomised trial were enrolled (figure 1). Randomisation was performed by the Trials Unit interactive voice recognition system to one of two groups: initial medical management or initial invasive management (online supplemental methods).

### Registry

Information was prospectively recorded in a registry for acute NSTE-ACS and prior CABG patients who were not randomised but consented to participate in the registry. Reasons for non-participation in the randomised trial were prospectively recorded: exclusion criteria present, unsuitability for either invasive or non-invasive management, physician preference, patient preference or a combination of these factors. Baseline and follow-up clinical information were obtained similarly to the trial patients.

### Follow-Up

Follow-up (via telephone contact, clinic visits, letter) with completion of quality of life assessments (EuroQol Visual Analogue Scale (EQ-VAS) and EuroQol 5 Dimensions 5 Levels (EQ-5D-5L)) was maintained 6 monthly until ≥18 months follow-up was reached for the final recruited patient (online supplemental methods).

### Clinical event committee

An independent clinical event committee reviewed the primary efficacy and safety endpoints (online supplemental methods).

### Outcomes

#### Primary outcome

Defined as postrandomisation rate of major adverse events (coprimary composite outcome), including one composite outcome for efficacy and one composite outcome for safety.

#### Primary efficacy outcome

Defined as all-cause mortality, rehospitalisation for refractory ischaemia/angina, MI or hospitalisation for HF. The endpoints were assessed during the study until the final randomised patient had completed 18 months follow-up.

#### Primary safety outcome

Defined as bleeding (Bleeding Academic Research Consortium (BARC) types 2–4),^22^ stroke, procedure-related MI (type 4a, universal definition), worsening renal function or haemodialysis during the index hospitalisation.

#### Secondary outcomes

1. Quality of life.
2. CCS angina class.
3. Hospitalisation for refractory ischaemia and/or angina.
4. Repeat invasive management during follow-up.
5. Freedom from coronary and/or bypass graft intervention.

### Definitions of adverse events

Refractory ischaemia, death, procedure-related MI, stroke, major bleeding and worsening renal function are defined in online supplemental methods.

## Results

Two hundred and seventeen patients with prior CABG and an unplanned hospitalisation for a suspected NSTE-ACS were screened (figure 1).

Eighty-four (39%) participants were identified during screening but were deemed ineligible for progressing into the trial or registry, of whom 24 (29%) did not consent and 60 (71%) consented but were ineligible (≥1 reason): 42 (70%) not confirmed NSTE-ACS, 39 (65%) not stabilised symptoms, 4 (7%) no prior CABG, 5 (8%) refractory ischaemia, 3 (5%) unable to provide informed consent and 22 (37%) unsuitable for invasive management.

Of 133 (61%) eligible patients who consented to either the trial or registry, 60 patients (mean±SD age, 71±9 years, 43 (72%) male) were randomised and 73 (mean±SD age, 72±10 years, 53 (73%) male) entered the registry. The decision for entering the registry included physician preference (58 (79%)), patient preference (28 (38%)) or both (15 (21%)).

### Baseline characteristics

The characteristics of the trial and registry participants are described (table 1). Compared with trial participants, registry patients were twice as likely to have valve disease (31 (42%) vs 12 (20%); p=0.01), a lower haemoglobin (mean±SD 127±18 vs 135±16 g/L; p=0.01), worse New York Heart Association class (37% vs 20% in class III or IV; p=0.01) and higher frailty index (27% vs 18% moderately or severely frail and 38% vs 23% mildly frail; p=0.03). Fifty (83%) trial and 59 (81%) registry patients participants had a previous left internal mammary artery graft (p=0.82). Baseline EQ-VAS, EQ-5D-5L and medications were similar.

**Table 1:** Baseline clinical characteristics of the trial and registry participants

Characteristics	Statistic	All N=133	Trial N=60	Registry N=73	P value
Clinical					
Age, years	Mean±SD	71±10	71±9	72±10	0.46
Female sex	N (%)	37 (28)	17 (28)	20 (27)	1.00
Obese (body mass index >30 kg/m^2^)	N (%)	37 (28)	22 (37)	15 (21)	0.05
Presentation type*					
Non-ST segment elevation MI	N (%)	90 (68)	41 (68)	49 (67)	1.00
Unstable angina	N (%)	43 (32)	19 (32)	24 (33)	1.00
Medical history					
Diabetes mellitus†	N (%)	50 (38)	21 (35)	29 (40)	0.60
Previous MI	N (%)	91 (68)	41 (68)	50 (68)	1.00
Cardiac arrhythmia	N (%)	47 (36)	19 (32)	28 (39)	0.37
Treated hypertension	N (%)	88 (66)	42 (70)	46 (63)	0.46
Renal impairment	N (%)	38 (29)	13 (22)	25 (34)	0.13
Peripheral vascular disease	N (%)	32 (24)	16 (27)	16 (22)	0.55
Cerebrovascular disease	N (%)	30 (23)	13 (22)	17 (23)	0.84
Congestive HF	N (%)	44 (33)	14 (23)	30 (41)	0.04
Anaemia	N (%)	20 (15)	5 (8)	15 (21)	0.05
Valve disease	N (%)	43 (32)	12 (20)	31 (42)	0.01
Pacemaker	N (%)	11 (8)	5 (8)	6 (8)	1.00
History of smoking					
Current	N (%)	27 (20)	12 (20)	15 (21)	0.35
Former (>3 months)	N (%)	80 (60)	33 (55)	47 (64)	
Never	N (%)	26 (20)	15 (25)	11 (15)	
Serum creatinine, μmol/L	Median (IQR)	86 (71–114)	84 (68–101)	89 (73–131)	0.11
Haemoglobin, g/L	Mean±SD	131±18	135±16	127±18	0.01
Charlson Comorbidity Index	Median (IQR)	5 (3–7)	4 (3–6)	5 (3–7)	0.28
Health-related quality of life, EuroQol 5 Dimensions 5 Levels score	Median (IQR)	0.674 (0.447–0.866)	0.748 (0.514–0.899)	0.658 (0.437–0.817)	0.11
ECG abnormalities at initial presentation					
ST-segment depression	N (%)	67 (50)	28 (47)	39 (53)	0.49
ST-segment elevation	N (%)	27 (20)	11 (18)	16 (22)	0.67
T-wave inversion	N (%)	89 (67)	38 (63)	51 (70)	0.46
Q-waves	N (%)	42 (32)	15 (25)	27 (37)	0.19
Left bundle branch block	N (%)	11 (8)	5 (8)	6 (8)	1.00
AF or flutter	N (%)	23 (17)	9 (15)	14 (19)	0.65
New ischaemic ECG changes‡	N (%)	66 (50)	30 (50)	36 (50)	1.00
Canadian Cardiovascular Society angina class§					
1	N (%)	4 (3)	2 (3)	2 (3)	0.83
2	N (%)	11 (8)	6 (10)	5 (7)	
3	N (%)	26 (20)	10 (17)	16 (22)	
4	N (%)	90 (69)	41 (69)	49 (68)	
New York Heart Association functional class					
I	N (%)	47 (35)	30 (50)	17 (23)	0.01
II	N (%)	47 (35)	18 (30)	29 (40)	
III	N (%)	28 (21)	8 (13)	20 (27)	
IV	N (%)	11 (8)	4 (7)	7 (10)	
Coronary artery bypass grafts					
Left internal mammary artery	N (%)	109 (82)	50 (83)	59 (81)	0.82
No/unknown	N (%)	24 (18)	10 (17)	14 (19)	
Saphenous vein graft					
0	N (%)	10 (8)	3 (5)	7 (10)	0.74
1	N (%)	37 (29)	17 (29)	20 (29)	
2	N (%)	54 (43)	25 (43)	29 (43)	
≥3	N (%)	25 (20)	13 (22)	12 (18)	
Frailty index					
Fit or well (1,2,3)	N (%)	60 (45)	35 (58)	25 (34)	0.03
Vulnerable (4) or mildly frail (5)	N (%)	42 (32)	14 (23)	28 (38)	
Moderately frail (6)	N (%)	29 (22)	10 (17)	19 (26)	
Severely frail (7)	N (%)	2 (2)	1 (2)	1 (1)	
Medication prior to hospital admission					
Aspirin	N (%)	113 (85)	52 (87)	61 (84)	0.81
Statin	N (%)	113 (85)	55 (92)	58 (79)	0.06
Beta-blocker	N (%)	94 (71)	42 (70)	52 (71)	1.00
Calcium channel blocker	N (%)	133 (100)	60 (100)	73 (100)	1.00
Isosorbide mononitrate	N (%)	50 (38)	20 (33)	30 (41)	0.38
Nicorandil	N (%)	47 (35)	22 (37)	25 (34)	0.86
ACE inhibitor	N (%)	101 (76)	50 (83)	51 (70)	0.10
Insulin	N (%)	20 (15)	10 (17)	10 (14)	0.64
Oral antidiabetic therapy	N (%)	28 (21)	10 (17)	18 (25)	0.29
Antidepressant therapy	N (%)	24 (18)	13 (22)	11 (15)	0.37
Diuretic	N (%)	52 (39)	18 (30)	34 (47)	0.07

Note that where there are missing values, the percentages are calculated out of the number of patients with data. Cardiac arrhythmia (2 missing from registry). New ischaemic ECG changes (1 missing from registry). Canadian Cardiovascular Society angina class (1 missing from trial, 1 missing from registry). Saphenous vein graft (2 missing from trial, 5 missing from registry).

Mean ± SD or median (IQR) for normal and non-normally distributed data, respectively.

*During the index hospitalisation, all patients in the randomised trial group had a type 1 MI, while seven patients in the registry group did not have a type 1 MI but had a type 2 MI (2 AF, 1 AF+HF, 1 severe aortic stenosis+HF, 1 anaemia, 1 AF+acute kidney injury, 1 HF+respiratory tract infection).

†Diabetes mellitus was defined as a history of diet-controlled or treated diabetes.

‡Any previous episode with new ischaemic ECG changes.

§The highest Canadian Cardiovascular Society value of any previous episode for each patient.

ACE, Angiotensin-converting enyzme; AF, atrial fibrillation; HF, heart failure; MI, myocardial infarction.

### In-hospital clinical course and invasive management

More than twice as many patients in the registry versus the trial had a medication change for recurrent angina. Approximately three-quarters of registry patients had medication changes for standard optimisation of secondary prevention therapy compared with trial patients (table 2).

**Table 2:** Reasons for changing medical therapy during the index hospitalisation

Reason	All N=133 (%)	Trial N=60 (%)	Registry N=73 (%)	P value
Recurrent angina	47 (35)	13 (22)	34 (47)	0.003
Standard optimisation of secondary prevention therapy	103 (77)	54 (90)	49 (67)	0.002
Intolerance of therapy without adverse reaction	8 (6)	2 (3)	6 (8)	0.293
Adverse drug reaction	10 (8)	2 (3)	8 (11)	0.113
Other	7 (5)	3 (5)	4 (5)	1.000

At baseline, invasive management (coronary angiography±PCI) was undertaken in 31 (52%) and 36 (49%) patients in the trial and registry groups, respectively, increasing during follow-up to 46 (77%) and 40 (55%), respectively (table 3). Of those who had invasive management at baseline and follow-up, PCI was performed in 10 (22%) and 13 (33%) of trial and registry patients at baseline, increasing to 21 (46%) and 18 (45%) patients during follow-up, respectively (table 3). For baseline procedures, the British Cardiovascular Intervention Society-1 Jeopardy Score was similar between trial (4±4) and registry (5±3; p=0.19) patients (table 3). At baseline and follow-up, compared with trial patients, registry patients had more urgent inpatient invasive procedures (39 (75%) vs 27 (47%)) and fewer outpatient invasive procedures (13 (25%) vs 30 (53%); p=0.004) (table 3). The culprit lesion was uncertain in 27 (47%) and 21 (40%) of procedures in the trial and registry groups, respectively (table 3). Adoption of adjunctive techniques, for example, rotational atherectomy was low (table 3).

**Table 3:** Invasive management (coronary angiography±PCI) in trial and registry patients at baseline (index admission) and follow-up (≥18 months)

Patients with procedures at baseline and follow-up	Trial N=46 (%)	Registry N=40 (%)	P value
Patients with one procedure	37 (80.4)	32 (80.0)	0.084
Patients with two procedures	8 (17.4)	4 (10.0)	
Patients with three procedures	0 (0)	4 (10.0)	
Patients with four procedures	1 (2.2)	0 (0)	
Patients with PCI at baseline	10 (21.7)	13 (32.5)	0.331
Patients with PCI at baseline and follow-up	21 (45.7)	18 (45.0)	1.000
**Days from enrolment to patient’s first procedure**	22.5 (6.5-64.75)	1.5 (0-7.25)	<0.001
<30 days	25 (54.3)	36 (90.0)	<0.001
30–59 days	9 (19.6)	0 (0)	
≥60 days	12 (26.1)	4 (10.0)	
**Procedures at baseline**	**Trial** **n=31**	**Registry** **n=36**	**P-value**
BCIS Jeopardy Score (pre-PCI) at baseline*	4.3±3.7	5.4±3.1	0.189
PCI at baseline	10 (32.3)	13 (36.1)	0.800
BCIS Jeopardy Score (post-PCI) at baseline	2.4±2.5	4.0±3.4	0.220
**Procedures at baseline and follow-up**	**Trial** **n=57**	**Registry** **n=52**	**P-value**
Urgent in-patient procedure	27 (47.4)	39 (75.0)	0.004
Outpatient procedure	30 (52.6)	13 (25.0)	
Hospitalisation†	28 (49.1)	41 (78.8)	0.002
Complications related to angiogram‡	1 (1.8)	6 (11.5)	0.052
Culprit vessel uncertain	27 (47.4)	21 (40.4)	0.563
Culprit vessel identified	30 (52.6)	31 (59.6)	
Graft only	17 (56.7)	15 (48.4)	0.611
Native artery only	12 (40.0)	15 (48.4)	0.609
Both graft and native artery	1 (3.3)	1 (3.2)	1.000
Multiple culprit lesions	3 (10.0)	2 (6.5)	0.671
PCI at baseline and follow-up	24 (42.1)	25 (48.1)	0.567
Thrombus aspiration	1 (4.2)	0 (0)	0.490
Rotational atherectomy	4 (16.7)	0 (0)	0.050
Intravascular ultrasound	2 (8.3)	3 (12.0)	1.000
Distal protection device	3 (12.5)	2 (8.0)	0.667

Mean±SD and median (IQR) for non-normally distributed data.

*BCIS Jeopardy score not available in one registry patient because of poor-quality angiogram and limited data for right coronary artery (only still frame and not a run).

†Admission to hospital including at least one overnight stay.

‡Complications in trial (N=1) was worsening renal function after angiography; complications in registry (N=6) were side branch abrupt closure, main branch distal embolisation into filter, no reflow, dissection post-angioplasty (+haematoma > 5 cm in same patient), pulmonary oedema on angiography table, side branch new/worsened thrombus.

BCIS, British Cardiovascular Intervention Society; PCI, percutaneous coronary intervention.

### Health outcomes

During a median of 694 (IQR 558–841) days follow-up, the primary efficacy outcome occurred in 26 (43%) and 36 (49%) trial and registry participants, respectively (HR 1.14, 95% CI 0.69 to 1.89) (table 4). The primary safety outcome occurred in 17 (28%) and 16 (22%) trial and registry participants, respectively (HR 0.74, 95% CI 0.37 to 1.46). The observed proportion of registry patients experiencing the efficacy outcome was slightly higher than in the trial group, whereas the opposite occurred for the safety outcome. When considered together (efficacy or safety), the rates were very similar between the groups. There was a lower proportion in the registry group experiencing both outcomes, but these events were very small in number.

**Table 4:** Primary and secondary outcomes over follow-up period (≥18 months; median 694 (IQR 558–841) days)

Outcome	Trial N=60	Registry N=73	HR (registry vs trial) (95% CI)
**Primary efficacy outcome**			
Composite of all-cause mortality, rehospitalisation for refractory ischaemia/angina, MI and HF	26 (43%)	36 (49%)	1.14 (0.69 to 1.89)
**Primary safety outcome**			
Composite of bleeding (BARC≥2), stroke, procedure-related MI and worsening renal function during the index hospitalisation	17 (28%)	16 (22%)	0.74 (0.37 to 1.46)
Experienced both primary efficacy and safety outcomes	10 (17%)	8 (11%)	0.64 (0.25 to 1.63)
Experienced either primary efficacy or safety outcomes	33 (55%)	44 (60%)	1.05 (0.67 to 1.65)
**Components of primary efficacy outcome**			
All-cause mortality	8 (13%)	17 (23%)	
Cardiovascular death	2 (3%)	10 (14%)	
Non-cardiovascular death	4 (7%)	5 (7%)	
Unknown cause of death	2 (3%)	2 (3%)	
Rehospitalisation for refractory ischaemia/angina	0 (0%)	0 (0%)	
Non-fatal MI	22 (37%)	18 (25%)	
HF	7 (12%)	11 (15%)	
**Primary efficacy outcome at 12 months**			
Composite of all-cause mortality, rehospitalisation for refractory ischaemia/angina, MI and HF at 12 months	20 (33%)	23 (32%)	
All-cause mortality at 12 months	5 (8%)	9 (12%)	
**Components of primary safety outcome**			
Bleeding (BARC 2–4)	11 (18%)	10 (14%)	
Stroke	0 (0%)	2 (3%)	
Procedure-related MI	0 (0%)	0 (0%)	
Worsening renal function during the index hospitalisation	8 (13%)	5 (7%)	
**Primary safety outcome at 12 months**			
Composite of bleeding (BARC≥2), stroke, procedure-related MI and worsening renal function during the index hospitalisation at 12 months	12 (20%)	14 (19%)	
**Other secondary outcomes**			
Total number of SAE	170	117	
Number of patients with a SAE	40 (67%)	49 (67%)	
Number of SAEs per patient, median (IQR)	1(0, 2)	1(0, 2)	
**Number of patients experiencing**			
Rehospitalisation	39 (65%)	47 (64%)	
Invasive management (coronary angiography)	46 (77%)	40 (55%)	
PCI	21 (35%)	18 (25%)	
Redo CABG	0 (-)	1 (1%)	
Coronary revascularisation (PCI or CABG)	21 (35%)	18 (25%)	
**Quality of life and angina**			
EQ-VAS health status, 6 months, median (IQR)	75 (60-80)	50 (40-75)	
EQ-5D-5L score, 6 months, median (IQR)	0.82 (0.53-0.94)	0.61 (0.29-0.82)	
CCS angina class, 6 months, median (IQR)	3.0 (1.0-3.0)	3.0 (2.0-4.0)	
EQ-VAS health status, 12 months, median (IQR)	70 (50-80)	58 (40-75)	
EQ-5D-5L score, 12 months, median (IQR)	0.82 (0.62-0.95)	0.78 (0.50-0.89)	
CCS angina class, 12 months, median (IQR)	3.0 (3.0-4.0)	3.0 (2.0-4.0)	

HRs and corresponding 95% CI from an unadjusted Cox model are given for the time from study entry to occurrence of primary outcomes only, comparing the registry to the trial group. Median (IQRs) are used for non-normally distributed data. Follow-up period was over median 694 (IQR 558–841) days.

BARC, Bleeding Academic Research Consortium; CABG, coronary artery bypass graft; CCS, Canadian Cardiovascular Society; EQ-5D-5L, EuroQol 5 Dimensions 5 Levels; EQ-VAS, EuroQol Visual Analogue Scale; HF, heart failure; MI, myocardial infarction; PCI, percutaneous coronary intervention; SAE, serious adverse event.

**Figure 2:**
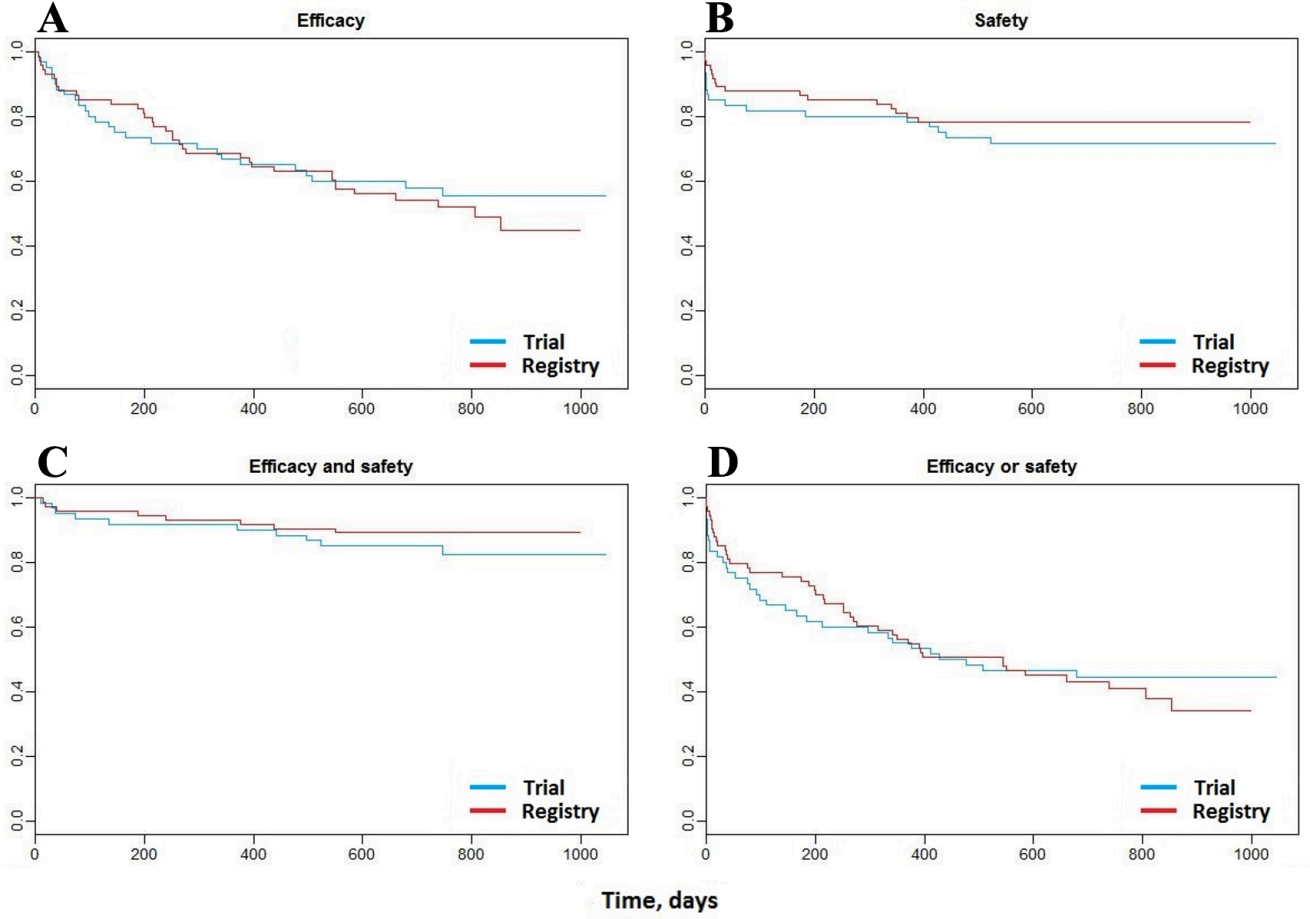
Kaplan-Meier survival curves: during a median of 694 (IQR 558–841) days follow-up: (A) The primary efficacy outcome occurred in 26 (43%) and 36 (49%) of trial and registry participants, respectively, HR 1.14, 95% CI 0.69 to 1.89. (B) The primary safety outcome occurred in 17 (28%) and 16 (22%) of trial and registry participants, respectively, HR 0.74, 95% CI 0.37 to 1.46. (C) The primary efficacy and safety outcome occurred in 10 (17%) and 8 (11%) of trial and registry participants, respectively, HR 0.64, 95% CI 0.25 to 1.63. (D) The primary efficacy or safety outcome occurred in 33 (55%) and 44 (60%) of trial and registry participants, respectively, HR 1.05, 95% CI 0.67 to 1.65.

Kaplan-Meier survival curves (figure 2) reveal that health outcomes in the trial and registry groups were similar during follow-up. Compared with the trial group, all-cause mortality and cardiovascular death occurred more often in the registry group, but non-fatal MI and worsening renal function during the index hospitalisation occurred less often (table 4). Two-thirds of patients in both groups experienced a serious adverse event (table 4). Redo CABG occurred in only one registry patient and in none of the trial participants (table 4).

### Health status

Compared to the trial group, the median EQ-VAS health status in the registry group was 25 points lower (worse) at 6 months, and 12 points lower at 12 months (table 4).

### Angina

The CCS angina class was similar between the groups at 6 months (median (IQR) trial 3 (1-3) vs registry 3 (2-4)) and 12 months (median (IQR) trial 3 (3-4) vs registry 3 (2-4)) (table 4).

## Discussion

The main findings of our study are, compared with the trial group, in the registry group: (1) multimorbidity, functional limitation, frailty and impaired health status were more pronounced; (2) changes to medication were more often made because of recurrent angina but less often made for standard optimisation of secondary prevention; (3) in invasively managed patients, the extent of jeopardised myocardium was similarly high and the culprit lesion was identified in half, and revascularisation by PCI was performed on one third; (4) health-related quality of life was lower at baseline, 6 and 12 months and (5) there was a fourfold increased risk of cardiovascular death, although power was limited. Overall, our study provides novel, contemporary insights into an understudied sub-group of ACS patients with a substantial health burden.

Our registry-based trial provided a framework for information to be prospectively gathered on patients who may have been eligible for randomisation but were not, including the reasons for not being randomised. Registry participation reflected physician and/or patient preferences for one form of treatment over another.^16^ These beliefs substantially limited enrolment into the randomised trial. This finding has implications for the design and funding of future trials in this population. Moreover, the finding in the comparison of the randomised trial groups that health outcomes were not different with invasive management versus conservative management supports the notion that enrolment rates could be increased by education of physicians and patients.^21^ Our trial results broadly reflect equipoise between the randomised strategies which should enhance confidence to support enrolment into a future randomised trial.

Compared with trial participation, registry participation was associated with a fourfold higher likelihood of cardiovascular death, with the caveat that event rates were very low for this outcome. This prognostic association may be partly explained by the greater burden of cardiovascular health problems at baseline, including HF and valve disease. Compared with trial patients, registry patients had more medication changes for recurrent angina—this may be partly explained by some registry patients having symptoms which were not stabilised and/or refractory ischaemia (both reasons for exclusion from the randomised trial) (figure 1). However, up-titration of medical therapy for secondary prevention occurred less often in the registry group, implying less intensive management, less scope for therapy improvements or both. The results highlight the substantial levels of morbidity, polypharmacy and adverse health outcomes in this group. The clinical course of two participants is illustrated (online supplemental figures 1 and 2).

10.1136/openhrt-2020-001453.supp2 Supplementary data

10.1136/openhrt-2020-001453.supp3 Supplementary data

### Advances in interventional management

In our trial, invasive management was selected in 36 (49%) of registry participants at baseline, but PCI was performed in only 13 (36%) of these patients. The lower PCI rate in our population may be explained by the complex nature of native vessel and graft disease, lack of a clear culprit (in almost half), and, arguably, lack of definitive evidence in support of the benefits of PCI in this population.

PCI continues to evolve with technical advances potentially leading to improvements in safety and procedural success (online supplemental discussion).

### Feasibility of a future substantive trial in patients with an NSTE-ACS and prior CABG

About one in 10–15 NSTE-ACS patients have a prior CABG (online supplemental table 1).^6^ This proportion is likely to remain stable in the coming years reflecting revascularisation practices in the past decade and increasing longevity. Many participants in this study were elderly, frail and multimorbid. Screening and obtaining informed consent were time-consuming for research staff. Medical decisions during urgent care may happen out-with office hours when research staff availability was limited. Medical information was commonly lacking at the time of hospitalisation, for example, graft history, limited recall by patients. These considerations present logistical barriers to enrolling patients into a randomised trial.

### High event rates in patients with an NSTE-ACS and prior CABG

Almost half of the participants in both groups experienced a primary efficacy outcome event (table 4). In contemporary trials involving NSTE-ACS patients, the 12-month major adverse cardiac event (MACE) rate is usually 8%–10%, which is very much lower than observed in this study’s patients. The proportion of affected patients increased substantially during longer-term follow-up beyond 12 months. Again, this progressive accrual of adverse cardiac events over time contrasts with other trials in NSTE-ACS patients in which cardiac events may plateau over time. The older age and universal presence of multi-morbidity probably explain the differences in prognosis between NSTE-ACS patients with versus without prior CABG. Considering future trials in NSTE-ACS patients with prior CABG, there are considerable logistical challenges to enrolment but, however, the event rate implies that the sample size may be lower than for other populations in which primary outcome event rates are expectedly lower.

### Limitations

Our pilot trial was not powered to assess for between-group differences in the rates of the serious adverse events contributing to the prespecified efficacy and safety outcomes. The sample size was small, with resultant wide confidence intervals. Both groups included patients that were managed differently (PCI vs medical therapy), thereby confounding between-group comparisons.

## Conclusion and potential value of results

Since clinical trials usually excluded patients with prior CABG, practice guidelines are not evidence based with respect to this group. In real-world practice, clinicians lack relevant information to inform decision making. Our trial and registry may be helpful in the design of clinical trials in this patient group.

## References

[1] Campbell PG , Teo KSL , Worthley SG , et al. Non-Invasive assessment of saphenous vein graft patency in asymptomatic patients. Br J Radiol 2009;82:291–5. 10.1259/bjr/19829466 19325046

[2] Tatoulis J , Buxton BF , Fuller JA . Patencies of 2127 arterial to coronary conduits over 15 years. Ann Thorac Surg 2004;77:93–101. 10.1016/S0003-4975(03)01331-6 14726042

[3] Cao C , Ang SC , Wolak K , et al. A meta-analysis of randomized controlled trials on mid-term angiographic outcomes for radial artery versus saphenous vein in coronary artery bypass graft surgery. Ann Cardiothorac Surg 2013;2:401–7. 10.3978/j.issn.2225-319X.2013.07.03 23977615 PMC3741882

[4] National Institute for Cardiovascular Outcomes Research (NICOR), Healthcare Quality Improvement Partnership (HQIP). National Adult Cardiac Surgery Audit: 2019 summary report, 2019. Available: https://www.hqip.org.uk/a-z-of-nca/heart-cardiac-surgery-audit-ncap/ [Accessed 17 Sep 2020].

[5] Bhatnagar P , Wickramasinghe K , Williams J , et al. The epidemiology of cardiovascular disease in the UK 2014. Heart 2015;101:1182–9. 10.1136/heartjnl-2015-307516 26041770 PMC4515998

[6] Shoaib A , Kinnaird T , Curzen N , et al. Outcomes following percutaneous coronary intervention in Non-ST-Segment-Elevation myocardial infarction patients with coronary artery bypass grafts. Circ Cardiovasc Interv 2018;11:e006824. 10.1161/CIRCINTERVENTIONS.118.006824 30571201

[7] Windecker S , Kolh P , et al, Authors/Task Force members. 2014 ESC/EACTS guidelines on myocardial revascularization: the Task Force on Myocardial Revascularization of the European Society of Cardiology (ESC) and the European Association for Cardio-Thoracic surgery (EACTS). Developed with the special contribution of the European Association of Percutaneous Cardiovascular Interventions (EAPCI). Eur Heart J 2014;35:2541–619. 10.1093/eurheartj/ehu278 25173339

[8] Hamm CW , Bassand JP , Agewall S , et al. ESC guidelines for the management of acute coronary syndromes in patients presenting without persistent ST-segment elevation: the task force for the management of acute coronary syndromes (ACS) in patients presenting without persistent ST-segment elevation of the European Society of Cardiology (ESC). Eur Heart J 2011;32:2999–3054. 10.1093/eurheartj/ehr236 21873419

[9] National Institute for Health and Care Excellence (NICE). Unstable angina and NSTEMI: early management. Clinical guideline 94, 2010. Available: https://www.nice.org.uk/guidance/cg94 [Accessed 17 Sep 2020]. 32065742

[10] Kim MS , Wang TY , Ou FS , et al. Association of prior coronary artery bypass graft surgery with quality of care of patients with non-ST-segment elevation myocardial infarction: a report from the National Cardiovascular Data Registry Acute Coronary Treatment and Intervention Outcomes Network Registry-Get With the Guidelines. Am Heart J 2010;160:951–7. 10.1016/j.ahj.2010.07.025 21095285

[11] Elbarasi E , Goodman SG , Yan RT , et al. Management patterns of non-ST segment elevation acute coronary syndromes in relation to prior coronary revascularization. Am Heart J 2010;159:40–6. 10.1016/j.ahj.2009.09.019 20102865

[12] Al-Aqeedi R , Asaad N , Al-Qahtani A , et al. Acute coronary syndrome in patients with prior coronary artery bypass surgery: observations from a 20-year registry in a Middle-Eastern country. PLoS One 2012;7:e40571. 10.1371/journal.pone.0040571 22815766 PMC3399890

[13] Nikolsky E , McLaurin BT , Cox DA , et al. Outcomes of patients with prior coronary artery bypass grafting and acute coronary syndromes: analysis from the ACUITY (Acute Catheterization and Urgent Intervention Triage Strategy) trial. JACC Cardiovasc Interv 2012;5:919–26. 10.1016/j.jcin.2012.06.009 22995879

[14] Fanning JP , Nyong J , Scott IA , et al. Routine invasive strategies versus selective invasive strategies for unstable angina and non-ST elevation myocardial infarction in the stent era. Cochrane Database Syst Rev 2016:CD004815. 10.1002/14651858.CD004815.pub4 27226069 PMC8568369

[15] Asrar Ul Haq M , Rudd N , Mian M , et al. Predictors and outcomes of early coronary angiography in patients with prior coronary artery bypass surgery presenting with non-ST elevation myocardial infarction. Open Heart 2014;1:e000059. 10.1136/openhrt-2014-000059 25332800 PMC4195928

[16] Feit F , Brooks MM , Sopko G , et al. Long-term clinical outcome in the Bypass Angioplasty Revascularization Investigation Registry: comparison with the randomized trial. BARI Investigators. Circulation 2000;101:2795–802. 10.1161/01.cir.101.24.2795 10859284

[17] Sanchis J , Núñez E , Barrabés JA , et al. Randomized comparison between the invasive and conservative strategies in comorbid elderly patients with non-ST elevation myocardial infarction. Eur J Intern Med 2016;35:89–94. 10.1016/j.ejim.2016.07.003 27423981

[18] de Belder A . Revascularisation or medical therapy in elderly patients with acute anginal syndromes (RINCAL), 2014. Available: https://clinicaltrials.gov/ct2/show/NCT02086019 [Accessed 17 Sep 2020]. 10.4244/EIJ-D-20-00975 PMC9724962 33226000

[19] Godfrey R , de Belder A . 162 recruitment for clinical trials for elderly patients – insights from the XIMA and RINCAL trials. Heart 2019;105:A135. 10.1136/heartjnl-2019-BCS.159

[20] Lee MMY , Petrie MC , Rocchiccioli P , et al. Non-invasive versus invasive management in patients with prior coronary artery bypass surgery with a non-ST segment elevation acute coronary syndrome: study design of the pilot randomised controlled trial and registry (CABG-ACS). Open Heart 2016;3:e000371. 10.1136/openhrt-2015-000371 27110377 PMC4838768

[21] Lee MMY , Petrie MC , Rocchiccioli P , et al. Invasive versus medical management in patients with prior coronary artery bypass surgery with a non-ST segment elevation acute coronary syndrome. Circ Cardiovasc Interv 2019;12:e007830. 10.1161/CIRCINTERVENTIONS.119.007830 31362541 PMC7664981

[22] Mehran R , Rao SV , Bhatt DL , et al. Standardized bleeding definitions for cardiovascular clinical trials: a consensus report from the Bleeding Academic Research Consortium. Circulation 2011;123:2736–47. 10.1161/CIRCULATIONAHA.110.009449 21670242

