# Supplementary material for: Invasive versus medically managed acute coronary syndromes with prior bypass (CABG-ACS): insights into the registry versus randomised trial populations
Source: Open Heart. 2021 Feb 26;8(1):e001453. doi: 10.1136/openhrt-2020-001453 (PMC7919592; doi:10.1136/openhrt-2020-001453)
Supplement: Supplementary data [file openhrt-2020-001453supp001.pdf]

SUPPLEMENTAL MATERIAL

Supplemental Methods

Setting

Study participants were enrolled in two large urban hospitals and two regional district general hospitals in the National Health Service (NHS) in the United Kingdom (UK). The hospitals differed in geography, availability of catheter laboratory facilities on-site (or not), and hospital type (academic vs. regional). Royal Blackburn Hospital was the only hospital with an on-site cardiac catheterisation laboratory. In the other hospitals, patients were triaged for invasive management by referral and transferred to the regional cardiothoracic centre (Golden Jubilee National Hospital).

Screening

The clinical research team on each site screened for patients aged ≥18 years, of either sex, admitted during unscheduled emergency care with a suspected acute non-ST segment elevation acute coronary syndrome and prior coronary artery bypass graft (CABG). Screening took place in the acute medical and cardiology wards during the course of routine healthcare. Patients eligible for either invasive (with coronary and graft angiography) or non-invasive management were invited to participate. Eligible patients were given an information sheet prior to participation. All randomised and registry patients provided written informed consent as soon as feasible after hospital admission and prior to referral for coronary angiography. Each patient was given a site and study number and entered into a screening log which only contained de-identified information. Patients who did not consent were included in the 'screen failure' log.

The community health index (CHI) or NHS number was recorded to enable electronic record linkage.

Randomisation

Randomisation was stratified by centre, using randomised permuted blocks of length 4 and 6, with block lengths chosen at random.

Non-invasive group

Participants who had been randomised to the non-invasive group could be referred for invasive management if any pre-specified criteria were met (**Supplemental Methods**).

Invasive group

Invasive management was performed early (i.e. ≤72 hours wherever possible) after hospital admission. Invasive management included native coronary and bypass graft angiography and coronary and/or graft revascularisation with percutaneous coronary intervention (PCI) and/or CABG, as clinically appropriate.

Optimal medical therapy

Optimal medical therapy was intended for all of the participants. Guidance on up-titration of medical therapy was provided in an investigator guideline. Medical therapy included dual anti-platelet, anti-thrombotic, and anti-ischaemic therapies as per local protocols and international guidelines.[1,2]

Non-invasive group

Study participants who had been randomised to the non-invasive group could be referred for invasive management if one of the following pre-specified criteria are met: 1) recurrent or

BMJ Publishing Group Limited (BMJ) disclaims all liability and responsibility arising from any reliance placed on this supplemental material which has been supplied by the author(s)

refractory (class III or IV) angina with documented ischaemic electrocardiogram (ECG) changes whilst on “optimal” anti-ischaemic therapy, 2) new ST-segment elevation in two contiguous leads without Q waves or T wave inversion greater than 3 mm or development of haemodynamic instability, or 3) a deterioration in heart failure (HF) status (consistent with Killip class 3 or 4) that the attending clinician judges to be ischaemia-related based on the presence of symptoms, ECG changes and cardiac biomarker elevation.

Follow-up and outcome collection

Clinical research nurses and clinicians who were independent of the study teams and aware of the group allocations supported enrolment and follow-up assessments on all sites. They prospectively gathered information on screening, recruitment, randomisation (to medical therapy or invasive management), crossover rates, and serious adverse events in patients with prior coronary artery bypass graft and a recent non-ST segment elevation acute coronary syndrome.

Data will be held for up to 20 years to enable long-term follow-up analyses. Following randomisation, clinical assessments involved gathering information from standard-of-care clinical reviews (end of hospitalisation, 30-42 days and 1 year) and also from clinical contacts recorded in the patients’ medical records. In West of Scotland hospitals, a single system of electronic patient records is used for all hospital attendances and correspondence with primary care.

Serious adverse events during the index admission and follow-up were evaluated from review of patient records obtained during usual care, and electronic health databases, using the CHI and NHS number. All outcomes were prospectively entered into an electronic Case Report Form.

Clinical Event Committee

The Clinical Event Committee (CEC) reviewed cases of interest to determine if they meet the criteria defined in the pre-specified charter. Causality assessments were not made by the CEC. The CEC was blinded to all information relating to the randomisation group. The CEC included 4 cardiovascular physicians who have expertise in the diagnosis and treatment of cardiovascular disorders and in the medical aspects of clinical trials. The CEC had a Chairman (M.C.P.) and coordinator (M.M.Y.L.) to assist with preparation of de-identified source clinical data, reports and communication with the Trials Unit. The CEC followed a pre-determined adjudication charter.

Definitions of adverse events

Refractory ischaemia

Recurrent ischaemic symptoms lasting more than 5 minutes, whilst on optimal medical therapy (at least 2 anti-anginal treatments) with documented characteristic ECG changes indicative of ischaemia and requiring an additional intervention. An additional intervention was defined as reperfusion therapy for myocardial infarction (MI), cardiac catheterisation, and insertion of intra-aortic balloon pump or revascularisation procedure (percutaneous coronary intervention or coronary artery bypass graft surgery) within 48 hours of the onset of this episode. This definition is in line with the Timing of Intervention in Acute Coronary Syndromes (TIMACS) trial.[3]

Death

All-cause, sudden cardiac death, death due to MI, death due to HF, death due to stroke, death due to extra-axial haemorrhage, death due to cardiovascular operation, death due to other

cardiovascular cause (e.g. infective endocarditis), presumed cardiovascular death (undetermined cause of death), non-cardiovascular death.[4]

Procedure-related MI

According to the Universal Definition of MI (Type 4b).[5] A post-procedure ECG was used to diagnose Q-wave vs. non-Q-wave MI.

Stroke

Defined as the presence of a new focal neurologic deficit thought to be vascular in origin, with signs or symptoms lasting more than 24 hours;[3] subdural haemorrhage.

Major bleeding

Defined according to the Bleeding Academic Research Consortium criteria.[6]

Worsening renal function

Defined as deterioration in estimated Glomerular Filtration Rate ≥ 25% of baseline during the index admission.

Crossover

A crossover between groups was defined as a change of treatment strategy from invasive to non-invasive management, or vice versa. In addition, we pre-defined crossover as occurring within 30 days after randomisation.

Sample Size

Since CABG-ACS was an exploratory pilot trial, no sample size calculation was performed. The sample size was n=60 based on the number of participants projected to be enrolled in 4 hospitals within a 12–18 month period. We chose this number across different secondary care settings to

be broadly representative of the diversity in UK hospitals. The sample size was selected to enable the feasibility of randomisation, and the reasons for not being randomised were prospectively recorded. The trial was designed but not powered to assess for between-group differences in the rates of the serious adverse events contributing to the prespecified efficacy and safety outcomes.

Data management and biostatistics

The Robertson Centre for Biostatistics acted as an independent coordinating centre for data management and statistical analyses. The Centre is registered with a Clinical Trials Unit (National Institute for Health Research Registration number: 16). The Chief Investigator (Professor Berry) had full access to all the data in the study and takes responsibility for its integrity and the data analysis.

Patient confidentiality

Patients were assigned an identification code at the time of recruitment.

Statistical analysis

Baseline data

Baseline characteristics were summarised using mean (standard deviation (SD)) or median (interquartile range (IQR) for skewed data) for continuous variables, and count (%) for categorical variables. Baseline characteristics for randomised vs. registry participants were compared using t-tests, Mann-Whitney tests and chi-squared tests (or Fisher's exact tests) as appropriate.

Efficacy and safety outcomes

Numbers of events and numbers (%) of patients with adverse events were summarised. The proportion of patients with adverse events was compared between the registry and trial groups with a chi-squared test. Kaplan-Meier curves were produced for time to occurrence of the primary efficacy and safety outcomes. The hazard ratios (HR) of the primary outcomes, comparing the registry to trial group, were calculated with corresponding 95% confidence interval (CI) from a Cox model. Secondary outcomes were presented as descriptive statistics only, since this was a pilot trial with insufficient power for statistical testing of these outcomes.

Ethics

Potential benefits to participants include avoidance of harmful invasive management and avoidance of longer-term stent failure. No additional interventions were proposed nor were procedures withdrawn that would be needed on clinical grounds. While the intention-to-treat in each group was either with non-invasive or invasive management, all treatment options remained available according to patient and physician preference i.e. patients initially randomised to medical therapy could have undergone invasive management and vice versa.

Trial management

A Trial Management Group including the researchers and Local Principal Investigator on each of the 4 sites coordinated the study's activities on a day-to-day basis. The NHS Sponsor monitored the trial. Since the trial was a pilot, there was no Independent Data and Safety Monitoring Committee.

Supplemental Discussion

Pivotal trials excluded patients with prior CABG, limiting the applicability of practice guidelines that recommend invasive management in non-ST segment elevation acute coronary syndrome (NSTE-ACS) with prior CABG.[1,2,7] Our results provide real-world insights into the baseline characteristics, treatment and outcomes of patients who were ineligible for randomisation but provided informed consent for registry participation. Commonly, this information is not gathered in clinical trials due to resource implications and logistics. Bypass Angioplasty Revascularization Investigation (BARI) was a trial of percutaneous transluminal coronary angioplasty (PTCA) versus CABG.[8] BARI included a registry of eligible patients who were not randomised based on physician and/or patient preference.[8] The main reason for not being randomised was physician and patient preference for PTCA. In BARI, the physicians selected PTCA rather than CABG for 65% of registry patients who underwent revascularisation without compromising long-term survival either in the overall population or in patients with treated diabetes. This result is in contrast to the randomised trial where patients with treated diabetes who underwent CABG gained a survival advantage compared to those patients with treated diabetes who had PTCA.

We also gathered information on the selection process for trial participation, providing insights into the reasons for this decision. Within the registry, invasive management was substantially the preferred strategy by physicians and cardiologists. However, the proportions of patients treated by PCI in the registry group and the invasive group in the randomised trial were similarly low. A registry can disclose information on patient subsets in whom an intervention may have differential effects (harm or benefit).

Registry patients who were selected for invasive management may have been identified by clinicians as being potentially amenable to gaining symptomatic or prognostic benefit from revascularisation. Conversely, registry patients who were selected for medical management may have been judged as having little to gain and at risk of harm from invasive management and with non-modifiable chronic health impairment.

Advances in interventional management

In recent years, radial artery access has become the standard approach for invasive management rather than femoral artery access. The left radial artery can provide arterial access in patients with a left internal mammary graft. However, Complete High risk Indicated Patient (CHIP) procedures may require simultaneous left and right coronary artery access which necessitates vascular access via the femoral artery. Overall, our results support the safety of invasive management in selected NSTE-ACS patients with prior CABG.

Advances in CHIP procedures lead to new possibilities for revascularisation in patients with complex disease.[9] Specialist techniques have developed with ‘antegrade’ and ‘retrograde’ approaches to recanalize chronic totally occluded (CTO) coronary arteries such that CTO PCI in native vessel CTOs has become increasingly feasible.[9] However, CTO PCI procedures are complex, require advanced skills, only undertaken by a minority of interventional cardiologists, and are usually pre-planned on an elective basis. Equipment can be expensive. Some of these techniques evolved very recently and so were not routinely implemented in the invasively managed patients. Whether CHIP procedures would increase revascularisation rates, comparable safety, and improvements in prognosis merit prospective evaluation in a substantive multicentre trial.[9]

Abbreviations: Acronyms of trials

FRISC II = FRagmin and Fast Revascularisation during InStability in Coronary artery disease

ICTUS = Invasive versus Conservative Treatment in Unstable Coronary Syndromes

ISAR-COOL = Intracoronary Stenting with Antithrombotic Regimen Cooling-Off

LIPSIA-NSTEMI = The Leipzig Immediate versus early and late PercutaneouS coronary Intervention triAl in NSTEMI

MATE = Medicine versus Angiography in Thrombolytic Exclusion

MOSCA = coMOrbilidades en el Síndrome Coronario Agudo

OASIS-5 = Fifth Organization to Assess Strategies in Ischemic Syndromes

RINCAL = Revascularisation or Medical Therapy in Elderly Patients with Acute Anginal Syndromes

RITA 3 = Randomized Intervention Trial of unstable Angina

TACTICS-TIMI 18 = Treat Angina with Aggrastat and Determine Cost of Therapy with an Invasive or Conservative Strategy – Thrombolysis in Myocardial Infarction 18

TIMACS = Timing of Intervention in Acute Coronary Syndromes

TIMI IIIB = Thrombolysis in Myocardial Ischemia

TRUCS = Treatment of Refractory Unstable angina in geographically isolated areas without Cardiac Surgery

VANQWISH = Veterans Affairs Non-Q-Wave Infarction Strategies in Hospital

VINO = Value of first day angiography/angioplasty In evolving Non-ST segment elevation myocardial infarction: an Open multicenter randomized trial

Supplemental Tables

Supplemental Table 1. Trials of patients with non-ST elevation acute coronary syndromes.

Trials which included patients with prior coronary artery bypass grafting (CABG)

Trial	Year published	N	N (%) with prior CABG
VANQWISH[10]	1998	920	156 (17.0%) (CABG >3 months before randomisation)
MATE[11]	1998	201	19 (9.5%)
TRUCS[12]	2000	148	18 (12.2%)
TACTICS-TIMI 18[13]	2001	2220	484 (21.8%) (CABG >6 months before randomisation)
ISAR-COOL[14]	2003	410	48 (11.7%)
ICTUS[15]	2005	1200	105 (8.8%)
OASIS-5[16]	2009	20078	1643 (8.2%)
Italian Elderly ACS[17]	2012	313	29 (9.3%)
LIPSIA-NSTEMI[18]	2012	600	41 (6.8%)
CABG-ACS pilot[19,20]	2016	60	60 (100.0%)
After Eighty study[21]	2016	457	76 (16.6%)
MOSCA[22]	2016	106	14 (13.2%)

Trials which excluded patients with prior CABG

Trial	Year published	N	Exclusion
TIMI IIIB[23]	1994	1473	CABG at any time
FRISC-II[24]	1999	2457	Previous open-heart surgery
VINO[25]	2002	131	CABG less than 6 months
RITA 3[26]	2002	1810	CABG at any time

Supplemental Figures

Supplemental Figure 1.

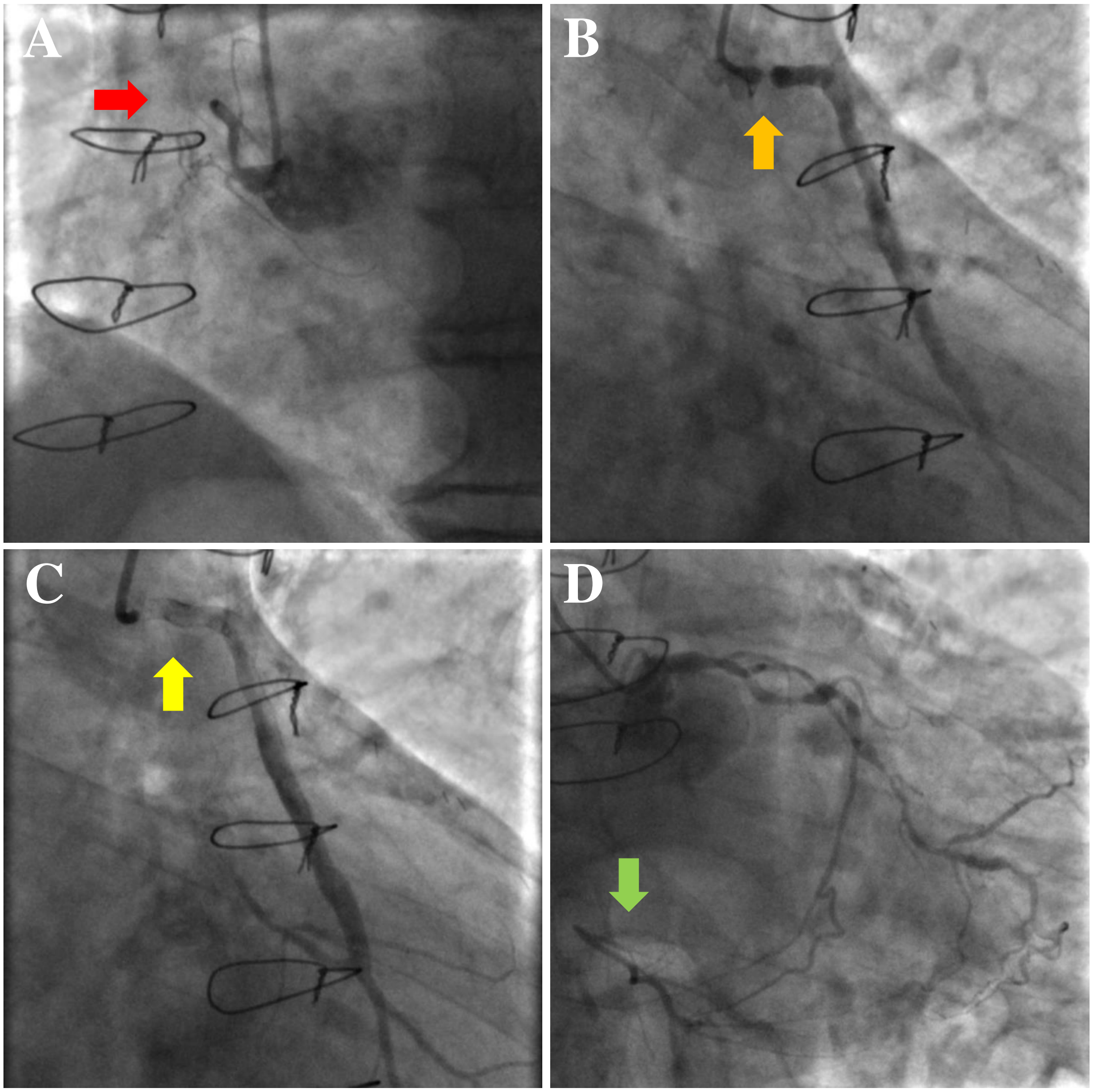

12

Supplemental Figure 2.

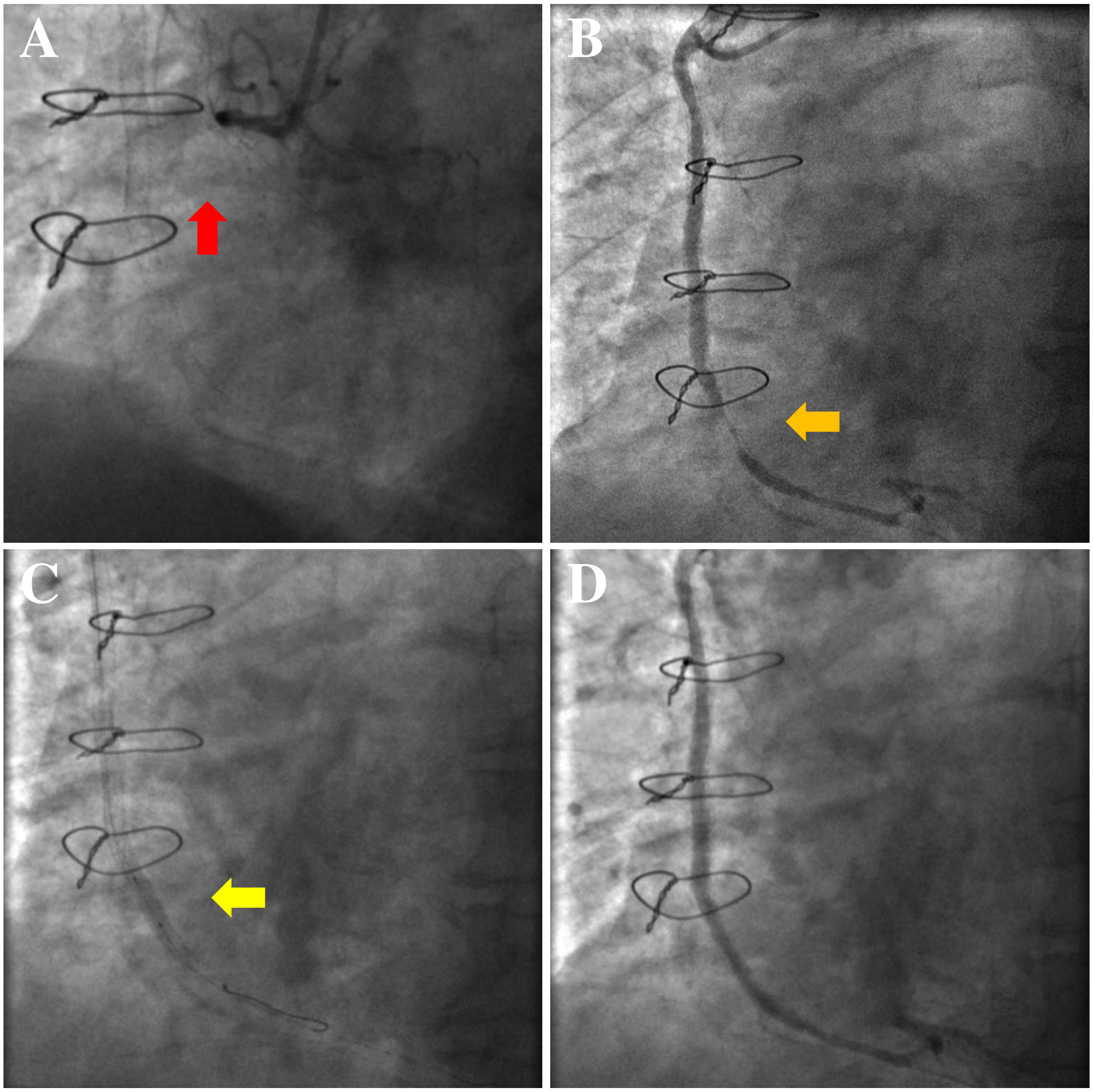

Lee MMY, *et al. Open Heart* 2021; 8:e001453. doi: 10.1136/openhrt-2020-001453

Supplemental Figure Legends

Supplemental Figure 1:

Clinical case example: This 72-year-old male in the registry had an inpatient coronary angiogram at baseline which showed a (A) blocked right coronary artery (red arrow) and a (B) tight stenosis in his saphenous vein graft supplying the obtuse marginal artery (orange arrow). He underwent (C) successful percutaneous coronary intervention to this saphenous vein graft (yellow arrow), thereby restoring flow to his (D) right coronary artery (green arrow) which was collateralised by his circumflex artery. This case highlights a diseased culprit vessel supplying collaterals to another territory that is not supplied by another patent saphenous vein graft or native artery. This participant experienced serious adverse events during follow-up including bleeding (Bleeding Academic Research Consortium type 2) and heart failure hospitalisation.

Supplemental Figure 2:

Clinical case example: This 63-year-old male with a history of previous coronary artery bypass grafting and multiple coronary stents for intractable angina, was admitted with a non-ST elevation acute coronary syndrome and was recruited to the CABG-ACS registry due to physician preference for invasive management. Urgent inpatient coronary angiography revealed a (A) occluded native right coronary artery (red arrow) and (B) diseased saphenous vein graft-right coronary artery (orange arrow). (C and D) Percutaneous coronary intervention to his saphenous vein graft-right posterior descending artery (yellow) was unsuccessful (plain old balloon angioplasty only, no stents). Following multi-disciplinary team discussion, he subsequently underwent redo coronary artery bypass grafting (long saphenous vein to posterior

descending artery) but unfortunately after a protracted post-operative recovery period, he did not survive.

Supplemental References

1 Windecker S, Kolh P, Alfonso F, *et al.* 2014 ESC/EACTS Guidelines on myocardial revascularization: The Task Force on Myocardial Revascularization of the European Society of Cardiology (ESC) and the European Association for Cardio-Thoracic Surgery (EACTS). *Eur Heart J* 2014;**35**:2541–619. doi:10.1093/eurheartj/ehu278

2 Hamm CW, Bassand JP, Agewall S, *et al.* ESC Guidelines for the management of acute coronary syndromes in patients presenting without persistent ST-segment elevation. *Eur Heart J* 2011;**32**:2999–3054. doi:10.1093/eurheartj/ehr236

3 Mehta SR, Granger CB, Boden WE, *et al.* Early versus delayed invasive intervention in acute coronary syndromes. *N Engl J Med* 2009;**360**:2165–75. doi:10.1056/NEJMoa0807986

4 Cutlip DE, Windecker S, Mehran R, *et al.* Clinical end points in coronary stent trials: a case for standardized definitions. *Circulation* 2007;**115**:2344–51. doi:10.1161/CIRCULATIONAHA.106.685313

5 Thygesen K, Alpert JS, Jaffe AS, *et al.* Third Universal Definition of Myocardial Infarction. *J Am Coll Cardiol* 2012;**60**:1581–98. doi:10.1016/j.jacc.2012.08.001

6 Mehran R, Rao S V., Bhatt DL, *et al.* Standardized bleeding definitions for cardiovascular clinical trials: a consensus report from the Bleeding Academic Research Consortium. *Circulation* 2011;**123**:2736–47. doi:10.1161/CIRCULATIONAHA.110.009449

7 NICE. Unstable angina and NSTEMI: early management. Clinical guideline 94. March 2010 (Updated November 2013). https://www.nice.org.uk/guidance/cg94 (accessed 17 Sep 2020).

8 Feit F, Brooks MM, Sopko G, *et al.* Long-term clinical outcome in the Bypass

Angioplasty Revascularization Investigation Registry: comparison with the randomized trial. BARI Investigators. *Circulation* 2000;**101**:2795–802. doi:10.1161/01.CIR.101.24.2795

9 Smith EJ, Strange JW, Hanratty CG, *et al.* Percutaneous intervention for chronic total occlusion: integrating strategies to address an unmet need. *Heart* 2013;**99**:1471–4. doi:10.1136/heartjnl-2013-304521

10 Boden WE, O'Rourke RA, Crawford MH, *et al.* Outcomes in patients with acute non-Q-wave myocardial infarction randomly assigned to an invasive as compared with a conservative management strategy. Veterans Affairs Non-Q-Wave Infarction Strategies in Hospital (VANQWISH) Trial Investigators. *N Engl J Med* 1998;**338**:1785–92. doi:10.1056/NEJM199806183382501

11 McCullough PA, O'Neill WW, Graham M, *et al.* A prospective randomized trial of triage angiography in acute coronary syndromes ineligible for thrombolytic therapy: Results of the medicine versus angiography in thrombolytic exclusion (MATE) trial. *J Am Coll Cardiol* 1998;**32**:596–605. doi:10.1016/S0735-1097(98)00284-8

12 Michalis LK, Stroumbis CS, Pappas K, *et al.* Treatment of Refractory Unstable angina in geographically isolated areas without Cardiac Surgery. Invasive versus conservative strategy (TRUCS Study). *Eur Heart J* 2000;**21**:1954–9. doi:10.1053/euhj.2000.2397

13 Cannon CP, Weintraub WS, Demopoulos LA, *et al.* Comparison of Early Invasive and Conservative Strategies in Patients with Unstable Coronary Syndromes Treated with the Glycoprotein IIb/IIIa Inhibitor Tirofiban. *N Engl J Med* 2001;**344**:1879–87. doi:10.1056/NEJM200106213442501

14 Neumann FJ, Kastrati A, Pogatsa-Murray G, *et al.* Evaluation of Prolonged Antithrombotic Pretreatment ("Cooling-Off" Strategy) Before Intervention in Patients

With Unstable Coronary Syndromes: A Randomized Controlled Trial. *JAMA* 2003;**290**:1593–9. doi:10.1001/jama.290.12.1593

15 de Winter RJ, Windhausen F, Cornel JH, *et al.* Early Invasive versus Selectively Invasive Management for Acute Coronary Syndromes. *N Engl J Med* 2005;**353**:1095–104. doi:10.1056/nejmoa044259

16 Jolly SS, Faxon DP, Fox KAA, *et al.* Efficacy and safety of fondaparinux versus enoxaparin in patients with acute coronary syndromes treated with glycoprotein IIb/IIIa inhibitors or thienopyridines: results from the OASIS 5 (Fifth Organization to Assess Strategies in Ischemic Syndromes) trial. *J Am Coll Cardiol* 2009;**54**:468–76. doi:10.1016/j.jacc.2009.03.062

17 Savonitto S, Cavallini C, Petronio AS, *et al.* Early Aggressive Versus Initially Conservative Treatment in Elderly Patients With Non–ST-Segment Elevation Acute Coronary Syndrome: A Randomized Controlled Trial. *JACC Cardiovasc Interv* 2012;**5**:906–16. doi:10.1016/j.jcin.2012.06.008

18 Thiele H, Rach J, Klein N, *et al.* Optimal timing of invasive angiography in stable non-ST-elevation myocardial infarction: The Leipzig Immediate versus early and late PercutaneouS coronary Intervention triAl in NSTEMI (LIPSIA-NSTEMI Trial). *Eur Heart J* 2012;**33**:2035–43. doi:10.1093/eurheartj/ehr418

19 Lee MMY, Petrie MC, Rocchiccioli P, *et al.* Non-invasive versus invasive management in patients with prior coronary artery bypass surgery with a non-ST segment elevation acute coronary syndrome: study design of the pilot randomised controlled trial and registry (CABG-ACS). *Open Heart* 2016;**3**:e000371. doi:10.1136/openhrt-2015-000371

20 Lee MMY, Petrie MC, Rocchiccioli P, *et al.* Invasive Versus Medical Management in Patients With Prior Coronary Artery Bypass Surgery With a Non-ST Segment Elevation

Acute Coronary Syndrome: A Pilot Randomized Controlled Trial. *Circ Cardiovasc Interv* 2019;**12**:e007830. doi:10.1161/CIRCINTERVENTIONS.119.007830

21 Tegn N, Abdelnoor M, Aaberge L, *et al.* Invasive versus conservative strategy in patients aged 80 years or older with non-ST-elevation myocardial infarction or unstable angina pectoris (After Eighty study): an open-label randomised controlled trial. *Lancet* 2016;**387**:1057–65. doi:10.1016/S0140-6736(15)01166-6

22 Sanchis J, Núñez E, Barrabés JA, *et al.* Randomized comparison between the invasive and conservative strategies in comorbid elderly patients with non-ST elevation myocardial infarction. *Eur J Intern Med* 2016;**35**:89–94. doi:10.1016/j.ejim.2016.07.003

23 The TIMI IIIB Investigators. Effects of tissue plasminogen activator and a comparison of early invasive and conservative strategies in unstable angina and non-Q-wave myocardial infarction. Results of the TIMI IIIB Trial. Thrombolysis in Myocardial Ischemia. *Circulation* 1994;**89**:1545–56. doi:10.1016/S0196-0644(94)70199-7

24 FRagmin and Fast Revascularisation during InStability in Coronary artery disease (FRISC II) Investigators. Invasive compared with non-invasive treatment in unstable coronary-artery disease: FRISC II prospective randomised multicentre study. *Lancet* 1999;**354**:708–15.

25 Spacek R, Widimský P, Straka Z, *et al.* Value of first day angiography/angioplasty In evolving Non-ST segment elevation myocardial infarction: an Open multicenter randomized trial: The VINO study. *Eur Heart J* 2002;**23**:230–8. doi:10.1053/euhj.2001.2735

26 Fox KAA, Poole-Wilson PA, Henderson RA, *et al.* Interventional versus conservative treatment for patients with unstable angina or non-ST-elevation myocardial infarction: the British Heart Foundation RITA 3 randomised trial. Randomized Intervention Trial of

unstable Angina. *Lancet* 2002;**360**:743–51. doi:10.1016/S0140-6736(02)09894-X
