# Supplementary figures and images for: Invasive versus medically managed acute coronary syndromes with prior bypass (CABG-ACS): insights into the registry versus randomised trial populations
Source: Open Heart. 2021 Feb 26;8(1):e001453. doi: 10.1136/openhrt-2020-001453 (PMC7919592; doi:10.1136/openhrt-2020-001453)

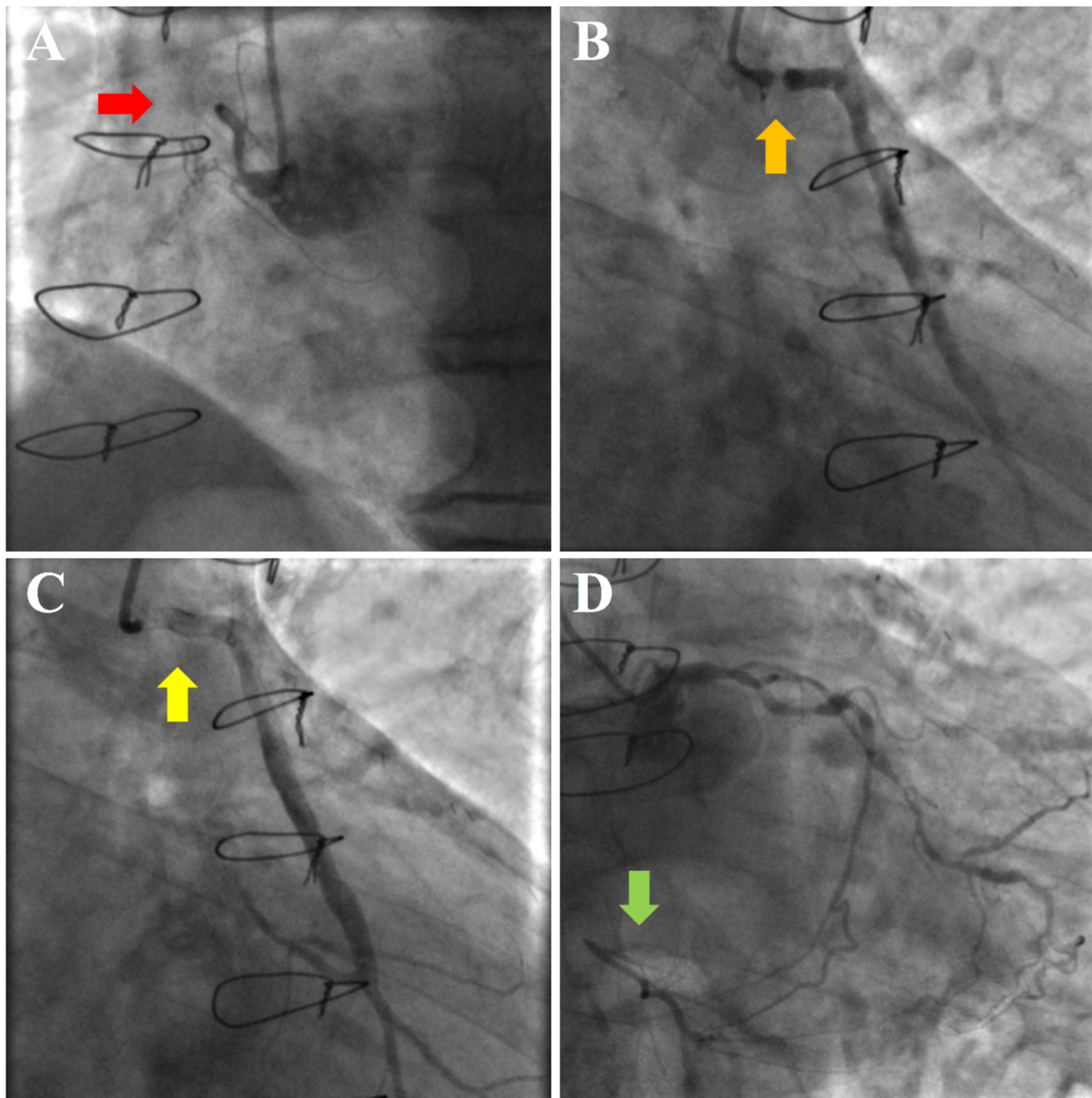

Supplement: Supplementary data [file openhrt-2020-001453supp002.pdf]

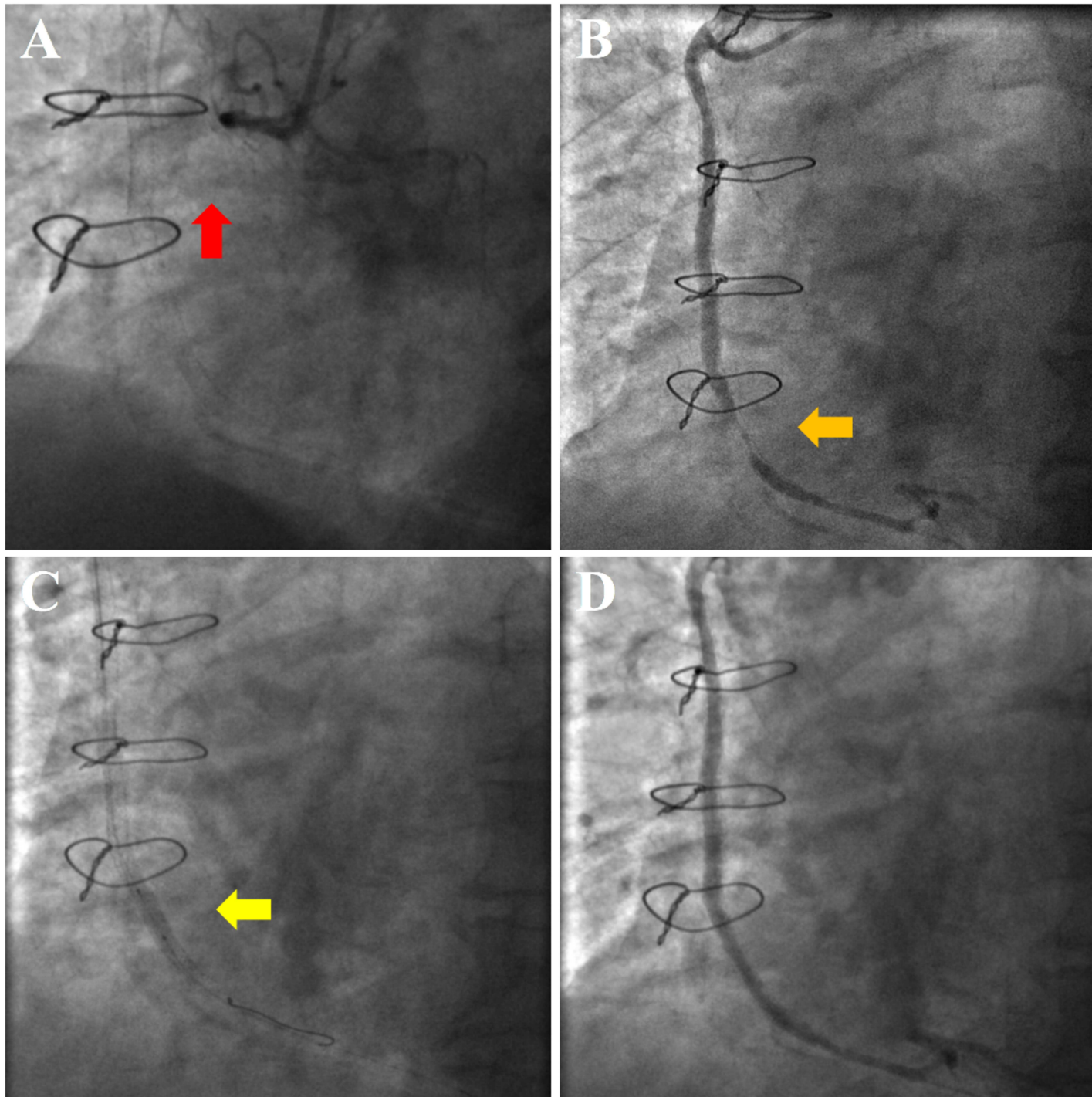

Supplement: Supplementary data [file openhrt-2020-001453supp003.pdf]
